# Transmission risk evaluation of transfusion blood containing low-density *Babesia microti*

**DOI:** 10.3389/fcimb.2024.1334426

**Published:** 2024-02-05

**Authors:** Yuchun Cai, Bin Xu, Xiufeng Liu, Wenwu Yang, Ziran Mo, Bin Zheng, Jiaxu Chen, Wei Hu

**Affiliations:** ^1^ Laboratory of Parasite and Vector Biology, Ministry of Public Health, World Health Organization (WHO) Collaborating Centre for Tropical Diseases, National Center for International Research on Tropical Diseases, Ministry of Science and Technology, Shanghai, China; ^2^ National Key Laboratory of Intelligent Tracking and Forecasting for Infectious Diseases, National Institute of Parasitic Diseases at Chinese Center for Disease Control and Prevention, Chinese Center for Tropical Diseases Research, Shanghai, China; ^3^ School of Life Sciences, Fudan University, Shanghai, China; ^4^ The institutes of Biomedical Sciences, College of Life Sciences, Inner Mongolia University, Hohhot, China

**Keywords:** human babesiosis, *Babesia microti*, transmission risk, transfusion transmission, detection limit

## Abstract

**Background:**

*Babesia* is a unique apicomplexan parasite that specifically invades and proliferates in red blood cells and can be transmitted via blood transfusion, resulting in transfusion-transmitted babesiosis. However, detecting *Babesia* in blood before transfusion has not received enough attention, and the risk of transfusing blood containing a low density of *Babesia microti* (*B. microti*) is unclear, possibly threatening public health and wellness.

**Purpose:**

This study aimed to determine the lower detection limit of *B. microti* in blood and to evaluate the transmission risk of blood transfusion containing low-density *B. microti*.

**Methods:**

Infected BALB/c mouse models were established by transfusing infected whole blood with different infection rates and densities of *B. microti*. Microscopic examination, nested Polymerase Chain Reaction (nested PCR), and an enzyme-linked immunosorbent assay (ELISA) were used to evaluate the infection status of the mouse models. Meanwhile, the nested PCR detection limit of *B. microti* was obtained using pure *B. microti* DNA samples with serial concentrations and whole blood samples with different densities of *B. microti*-infected red blood cells. Thereafter, whole mouse blood with a *B. microti* density lower than that of the nested PCR detection limit and human blood samples infected with *B. microti* were transfused into healthy mice to assess the transmission risk in mouse models. The infection status of these mice was evaluated through microscopic examination, nested PCR tests, and ELISA.

**Results:**

The mice inoculated with different densities of *B. microti* reached the peak infection rate on different days. Overall, the higher the blood *B. microti* density was, the earlier the peak infection rate was reached. The levels of specific antibodies against *B. microti* in the blood of the infected mice increased sharply during the first 30 days of infection, reaching a peak level at 60 days post-infection, and maintaining a high level thereafter. The nested PCR detection limits of *B. microti* DNA and parasite density were 3 fg and 5.48 parasites/μL, respectively. The whole blood containing an extremely low density of *B. microti* and human blood samples infected with *B. microti* could infect mice, confirming the transmission risk of transfusing blood with low-density *B. microti*.

**Conclusion:**

Whole blood containing extremely low density of *B. microti* poses a high transmission risk when transfused between mice and mice or human and mice, suggesting that *Babesia* detection should be considered by governments, hospitals, and disease prevention and control centers as a mandatory test before blood donation or transfusion.

## 1 Introduction

*Babesia* is a blood protozoan that parasitizes red blood cells. It belongs to the kingdom Protozoa, phylum Apicomplexa, class Porozoasida, subclass Piroplasmia, order Piroplasmid, family Babesiida, and genus *Babesia* (Homer et al., 2000; Froberg et al., 2004). *Babesia* is a unique apicomplexan parasite that specifically invades and proliferates in red blood cells. More than 100 species of *Babesia* have been isolated from wild animals and livestock, and those mainly infecting humans are *Babesia microti*, *B. divergens*, *B. duncani*, *B. venatorum*, and others, among which *B. microti* infection is the most common (Filbin et al., 2001; Conrad et al., 2006; Jiang et al., 2015).

Since the first case of human babesiosis in Yugoslavia in 1957, several reports have emerged worldwide, including reports of human babesiosis in Europe, Asia, Africa, North and South America, and Australia (Herwaldt et al., 2003). For example, since *Babesia* infection was reported as an infectious disease in the United States in 2011, approximately 1,000 - 2000 human cases have been reported annually (Gray and Herwaldt, 2019). According to a report from the Centers for Disease Control and Prevention, USA, in 2023, 2418 human cases of *Babesia* infections were reported in 2019, which was the highest recorded number since 2011. In 2020, 1827 human cases were recorded (Megan, 2020). *B. microti* infection is the most prevalent among humans (Moritz et al., 2016). Approximately 300 cases have been reported in China (Chen et al., 2020) in areas such as Heilongjiang, Yunnan, Chongqing, Guangxi, Shanghai, Xinjiang, Zhejiang, Inner Mongolia, Shandong, and Taiwan provinces. Among these, Heilongjiang and Yunnan have the most reported cases. The species distributed in China are mainly *B. microti*, *B. divergens* and *B. venatorum*, with *B. microti* being common in the south and *B. venatorum* being prevalent in the north (Qi et al., 2011). Among the cases reported in China, a few patients had a clear history of tick bites and surgical blood transfusions, but most patients had no clear infection mode.

*Babesia* infection of red blood cells results in lysis of the infected red blood cells, thus leading to hemolytic anemia. In addition to tick-mediated transmission, *Babesia* can also be transmitted via the transfusion of infected blood and blood products, resulting in transfusion-transmitted babesiosis (TTB) (Levin and Krause, 2016). In clinical practice, blood components are often transfused into patients for various purposes: 1) to meet the needs of the disease, 2) to reduce blood transfusion reactions and improve the safety of blood transfusion, and 3) to facilitate the preservation and use of blood components. If blood containing *Babesia* is infused into the human body, it will cause varying degrees of clinical reactions depending on the organism and dosage (Eberhard et al., 1995). It has been reported that intraperitoneal injection of 30 *Babesia* sporozoites could infect approximately 2/5 of inoculated hamsters, while 300 sporozoites could infect all the inoculated mice (5/5) (Etkind et al., 1980). Clinically, there are several cases of *Babesia* infection from blood transfusion. However, blood donors with extremely low-density of *Babesia* may not have obvious clinical symptoms, and if their blood is donated and directly transfused into patients without screening for *Babesia*, it is likely to cause symptoms and threaten the patient’s health. This is because the patient’s immune system is relatively weak and cannot suppress the growth of parasites. Unfortunately, the importance of detecting *Babesia* in donated blood products before transfusion has not received enough attention, and no licensed tests are currently required for screening *Babesia* in blood donation clinics. Therefore, the transmission risk of transfusion blood containing *B. microti*, especially those containing extremely low-density *B. microti*, is unknown.

Thus, this study aimed to evaluate the transmission risk of transfusing whole blood containing extremely low-density *B. microti.* The infection of mice inoculated with different densities *of B. microti* was tested, and then the transmission risk of transfusing whole blood containing an extremely low density of *B. microti* was evaluated by transfusing the infected whole blood between mice/mice and human/mice.

## 2 Materials and methods

### 2.1 Experimental animals

Six-week-old female BALB/c and NOD-SCID mice weighing approximately 20 g each were purchased from the Shanghai Experimental Animal Center at the Chinese Academy of Sciences [animal license number: SCXK (Shanghai) 2012-002]. All animal experiments conducted in this study were approved by the Experimental Animal Welfare and Ethics Committee of the Animal Center of the Institute of Parasitic Disease Prevention and Control and the Chinese Center for Disease Control and Prevention (Chinese Center for Tropical Diseases Research) (Animal Ethics Approval No: IPD-2019-14).

### 2.2 *Babesia* strain and infected whole blood sample

The standard *B. microti* Peabodymjr strain (ATCC, PRA-99) was purchased from the American Type Culture Collection (Manassas, VA) and maintained in BALB/c mice by serial passages, according to the methodology described previously (Cai et al., 2018). BALB/c mice were infected with the Peabodymjr strain and euthanized at seven days post-infection (dpi) to obtain anticoagulant whole blood for later use.

### 2.3 Human blood samples

Blood samples (557), including whole-blood (372) and red-cell samples (185), were obtained from the Health Examination Center of Shanghai, China. This study was approved by the Medical Ethics Committee of the National Institute of Parasitic Diseases and the Chinese Center for Disease Control and Prevention (Chinese Center for Tropical Diseases Research) (Ethical approval number: 2019004). Informed consent was obtained from the subjects or their family members for sample collection.

### 2.4 Infection analysis of the mice inoculated with different densities of *B. microti*

BALB/c mice were inoculated with *B. microti* by transfusing 100 μL of infected whole blood with infection rates of 20% (Group 1), 4% (Group 2), 0.8% (Group 3), 0.16% (Group 4), 0.032% (Group 5), and 0.0064% (Group 6). There were five mice in Group 1 and three mice in Groups 2-6. Whole blood (50-100 μL of blood from each mouse) was collected from the tail tips of the inoculated mice after 0, 2, 6, 7, 8, 10, 14, 21, 30, 45, 60, 120, 150, and 270 dpi. *Babesia microti* was detected in the blood samples from the six groups using microscopy(2.4.1), nested polymerase chain reaction (Nested PCR) (2.4.2) and enzyme-linked immunosorbent assay (ELISA) (2.4.3). These methods were also used to detect the infection status of mice in the transmission risk evaluation studies. In addition, the nested PCR detection limit of *B. microti* in whole blood was obtained according to the method described in section 2.4.2.

#### 2.4.1 Microscopy determination of *B. microti* infection rate of inoculated mice

Firstly, bright field microscopy was used to observe *B. microti* infection in the collected whole blood samples after preparing a thin blood smear and staining the samples with Giemsa staining. During the observation, 1000 red blood cells were selected per slide, from which the number of infected red blood cells was counted. The infection rate was then calculated using the following equation: Infection rate (%) = (number of infected red blood cells/1000) × 100%. Three slides were observed for each group, and the infection rates of the three groups were averaged to obtain the infection rate for the sample at that time point. Blood smears with a low density of *B. microti* were just observed without counting the *B. microti*.

#### 2.4.2 Nested PCR detection of *B. microti* nucleic acid in the blood of inoculated mice and the detection limit for *B. microti*

Nested PCR was used to detect the *B. microti* nucleic acid in the whole blood samples collected at different time points. Briefly, the DNA of *B. microti* was extracted from blood samples using a blood & tissue nucleic acid extraction kit (Qiagen Company, Germany), according to the manufacturer’s instructions. The nucleotide sequence of the 18S rRNA-specific fragment was amplified using the extracted DNA as the template, and nested PCR was performed to investigate the infection status of *B. microti* in the samples. External primers (CRYPTORN: 5’-GAATGATCCTTCCGCAGGTTCACCTAC-3’ and CRYPTOFL: 5’-AACCTGGTTGATCCTGCCAGTAGTCAT-3’) and internal primers (Bab1: 5’- GTCTTAGTATAAGCTTTTATACAGCG-3’ and Bab4: 5’- GATAGGTCAGAAACTTGAATGATACATCG-3’) were used for nested PCR, according to previously described protocols (Welc-Faleciak et al., 2007). The length of the nested PCR product was 238 bp.

To determine the nested PCR detection limit for *B. microti*, we used serial concentrations of pure *B. microti* DNA samples. Briefly, *B. microti* DNA (30 ng/μL) was diluted to a one-half ratio with double distilled water (dd H_2_O) ratio to obtain 11 DNA samples which were used as templates for the nested PCR detection. Meanwhile, whole blood collected from the inoculated mice was used to determine the nested PCR detection limit of *B. microti* density in blood. Briefly, whole blood with an infection rate of 20% (2.14 ×10^12^ *B. microti* infected red blood cells/L/10.71× 10^12^ red blood cells/L) was diluted at a 1/5-fold dilution ratio with normal BALB/c mouse whole blood to obtain 11 different whole blood samples, with the lowest concentration of 1.09 *B. microti* infected blood cells/μL. The DNA of *B. microti* was then extracted using these samples and used as templates for the nested PCR detection. The nested PCR detection limit of *B. microti* density in blood samples was obtained based on the relationship between the DNA and the density of *B. microti*.

#### 2.4.3 ELISA for quantifying specific anti-*B. microti* antibodies in serum

To quantify the specific anti-*B. microti* antibodies in the serum of the whole blood collected from the *B. microti-*inoculated mice at different time points, we performed serological analysis using enzyme-linked immunosorbent assay (ELISA). Briefly, the collected whole blood was allowed to clot by leaving it undisturbed at room temperature for 30 min. Blood clots were then collected by centrifuging the samples at a speed of 3000 r/min (with a centrifugation radius of 13.5 cm) in a refrigerated centrifuge for 10 min, and the resulting supernatant was designated serum. After that, the blood serum was subjected to ELISA to quantify the anti-*B. microti* antibody using a homemade ELISA kit. The wells of the ELISA plate were coated with an antigen of the recombinant protein rBm2D33 (Xu et al., 2018) (patent number 5283094). In addition to the ELISA kits, reagents used for ELISA were purchased from Jei Daniel (JD) Biotech Corp., China.

### 2.5 Transmission risk assessment of transfusing blood containing extremely low-density *B. microti*

#### 2.5.1 Assessment of mouse blood samples using mouse models

Whole blood samples containing a lower *B. microti* density (1.09 parasites/μL) than the nested PCR detection limit were used to assess the transmission risk of transfusing infected blood with extremely low *B. microti* density. The whole blood samples were inoculated into three healthy mice by transfusing 100 μL of the samples into each mouse. On the 14th and 28th dpi, the infection status of the inoculated mice was observed using microscopy (2.4.1) and the nested PCR test (2.4.2) to determine whether there was a risk of *B. microti* transmission during the transfusion of the blood containing an extremely low density of the parasite. On 0, 7, 14, and 28 dpi, the level changes of specific antibodies against *B. microti* were quantified in the serum using the ELISA test (2.4.3).

Meanwhile, the whole blood of the mice inoculated with the blood containing extremely low-density *B. microti* (Group 6, initial infection rate of 0.0064) for 120 days was confirmed to be *B. microti* negative using microscopy and nested PCR test. The whole blood collected from these mice was transfused to six healthy mice (second generation) at the amount of 100 µL/mouse. At 12 and 28 dpi, the infection status of the mice was observed using microscopy (2.4.1) and the nested PCR test (2.4.2) to determine whether there was a risk of *B. microti* transmission during the transfusion of the whole blood collected from the inoculated mice (first generation). After 0, 14, 28, and 60 dpi, the level changes of specific antibodies against *B. microti* were quantified in the serum using the ELISA test (2.4.3).

#### 2.5.2 Assessment of human blood samples using mouse models

Blood samples (557) were used to quantify the *B. microti* DNA using nested PCR (2.4.2). Of these 557 whole blood samples, 372 were used to quantify the specific anti-*B. microti* antibody using ELISA (2.4.3). Two *B. microti*-positive blood samples (samples 118 and 146) were identified by nested PCR, and six *B. microti*-positive blood samples, including sample 130, were identified by ELISA. After that, three BALB/c and two NOD-SCID mice were inoculated with the positive blood of sample 118 at the amount of 100 μL/mouse and were observed for 98 days. The infection status of these inoculated mice was evaluated at different pre-determined time points using microscopy and nested PCR. At the end of observation (98 days), the whole blood samples from the first-generation NOD-SCID mice that tested *B. microti* positive were inoculated into healthy NOD-SCID mice (the second generation) by blood transfusion and the infection status of the second-generation NOD-SCID mice was assessed for 65 days using microscopy and nested PCR. Moreover, to test the transmission risk of the positive blood sample130, we inoculated three BALB/c and three NOD-SCID mice at the amount of 100 μL/mouse. At the end of observation (98 days), the whole blood of the first generation of NOD-SCID mice that tested positive *B. microti* was inoculated into healthy NOD-SCID mice (the second generation) by blood transfusion. Additionally, the infection status of the second generation of NOD-SCID mice was assessed for 65 days using microscopy and nested PCR.

## 3 Results

### 3.1 Infection status of mice inoculated with different densities of *B. microti*

The mice were inoculated with different densities of *B. microti* and observed for 270 days. Microscopy examination was performed on the blood samples collected from the mice at different dpi, and the number of infected mice in each group at different dpi is shown in **Table 1**. The results indicated that increasing the infection rate of the transfused blood could cause rapid infection of the inoculated mice. Specifically, when the infection rate of the transfused blood was 20% (Group 1), all inoculated mice were infected from 2 dpi, but no infection was observed in other groups at the same time point. At 5 dpi, all mice in Groups 1 and 2 (with an infection rate of transfused blood of 4%) and two out of three mice (2/3) in Group 3 (with an infection rate of transfused blood of 0.8%) were infected, while those in other groups were not infected. Moreover, at 7 dpi, all mice in Groups 1, 2, and 3 were infected, while those in other groups were not. All mice in Groups 1, 2, and 3 and two out of three mice (2/3) in Group 4 (with an infection rate of transfused blood of 0.16%) were infected at 8 dpi, but those in Groups 5 and 6 were negative for *B. microti.* At 9 dpi, all mice in groups 1-5 and two out of three mice (2/3) in Group 6 were infected; thus, only one mouse in Group 6 was negative for *B. microti.* However, at 10 dpi, all mice in all groups were infected and remained positive for the next 4 days. After 14 dpi, the number of infected mice started to decrease. Specifically, at 30 dpi, *B. microti* was only detected in the blood samples collected from two mice (2/5) in Group 1 and one mouse in Group 2, and no mice were infected with *B. microti* in the other groups. The infection rate kept decreasing, and no *B. microti* parasites were found among the mice in any of the groups from 45 dpi until the end of the experiment (270 dpi).

**Table 1:** Detection of *Babesia microti* infection using Giemsa-stained thin blood smears of BALB/C mice during a 9-month study.

Microscopy (No. positive / No. tested)						
Dpi	Group 1 (20%; 5 mice)	Group 2 (4%; 3 mice)	Group 3 (0.8%; 3 mice)	Group 4 (0.16%; 3 mice)	Group 5 (0.032%; 3 mice)	Group 6 (0.0064%; 3 mice)
0						
2	√(5/5)					
3	√(5/5)					
5	√(5/5)	√(3/3)	√(2/3)			
6	√(5/5)	√(3/3)	√(2/3)			
7	√(5/5)	√(3/3)	√(3/3)			
8	√(5/5)	√(3/3)	√(3/3)	√(2/3)		
9	√(5/5)	√(3/3)	√(3/3)	√(3/3)	√(3/3)	√(2/3)
10	√(5/5)	√(3/3)	√(3/3)	√(3/3)	√(3/3)	√(3/3)
14	√(5/5)	√(3/3)	√(3/3)	√(3/3)	√(3/3)	√(3/3)
21	√(5/5)	x (0/3)	√(2/3)	√(1/3)	√(3/3)	√(3/3)
30	√(2/5)	√(1/3)	x (0/3)	x (0/3)	x (0/3)	x (0/3)
45	x (0/5)	x (0/3)	x (0/3)	x (0/3)	x (0/3)	x (0/3)
60	x (0/5)	x (0/3)	x (0/3)	x (0/3)	x (0/3)	x (0/3)
120	x (0/5)	x (0/3)	x (0/3)	x (0/3)	x (0/3)	x (0/3)
150	x (0/5)	x (0/3)	x (0/3)	x (0/3)	x (0/3)	x (0/3)
270	x (0/5)	x (0/2)	x (0/3)	x (0/3)	x (0/3)	x (0/3)

**Figure 1:**
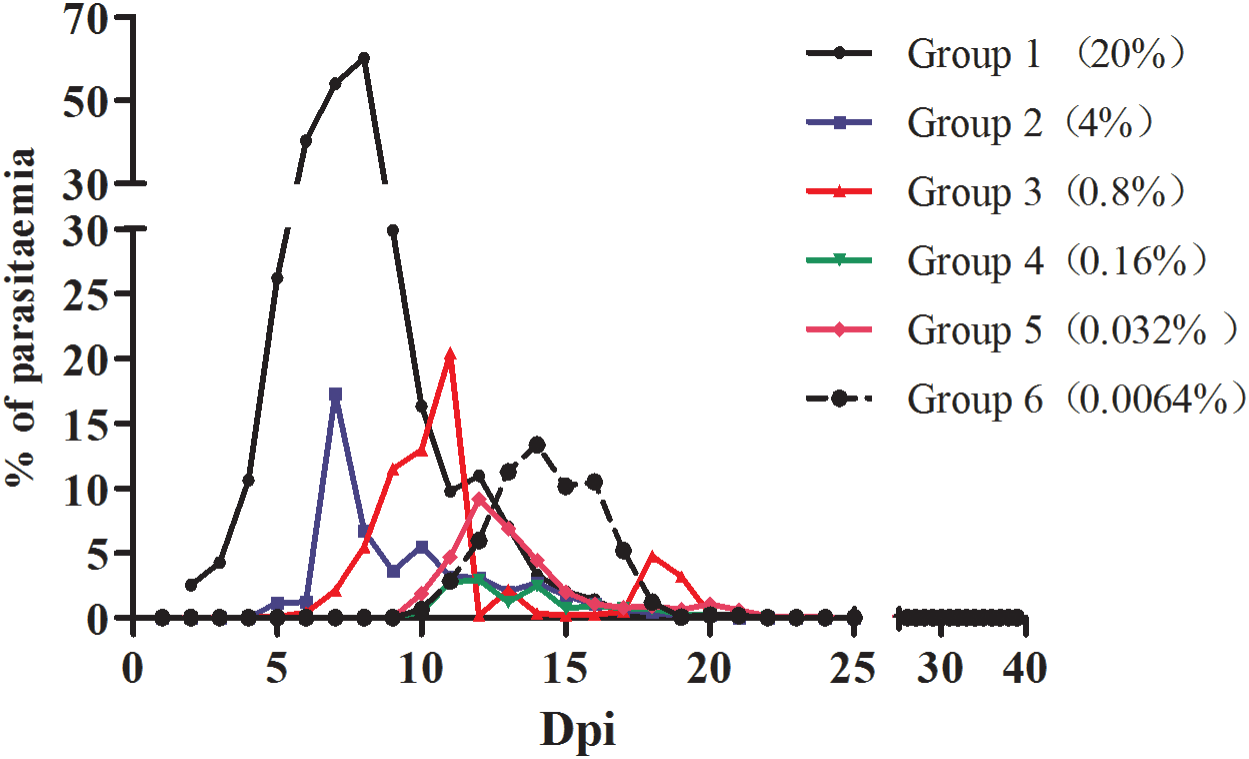
The infection rate of mice inoculated with blood containing different densities of *B. microti.* The infection rate of mice in different groups was calculated based on microscopy.

The infection rate of the mice was calculated for each group based on the microscopy examination results. As shown in **Figure 1**, mice inoculated with different densities of *B. microti* reached the highest infection rate (the infection peak) at different dpi. Specifically, Groups 1 and 2 reached the peak of infection rate at 7 dpi, Group 3 at 10 dpi, Groups 4 and 5 at 12 dpi, and Group 6 at 14 dpi.

**Figure 2:**
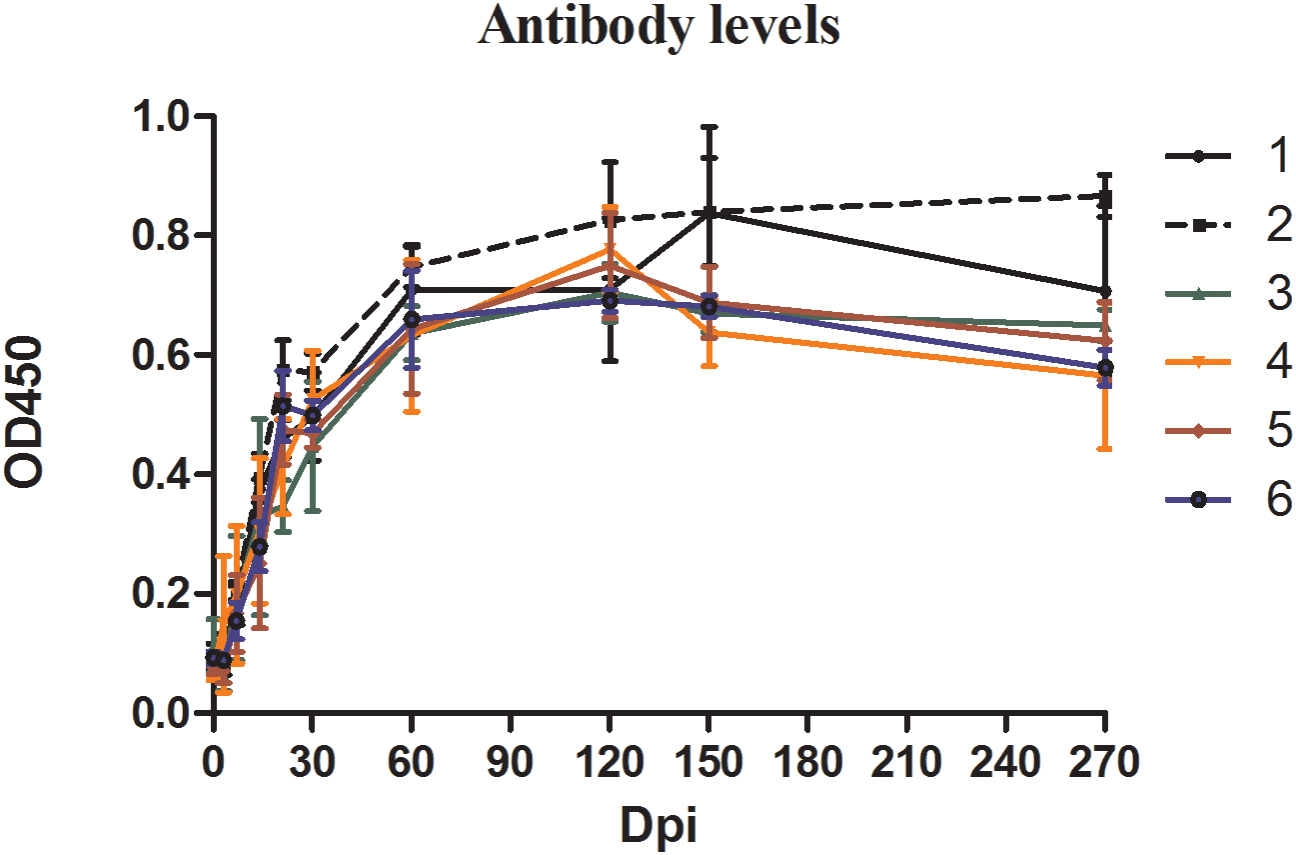
The antibody levels in mice inoculated with blood containing different densities of *Babesia microti.* The levels of specific antibodies against *B. microti* produced in mice at different time points in different groups were detected by enzyme-linked immunosorbent assay (ELISA).

ELISA was used to quantify the specific anti-*B. microti* antibody in the serum of the blood collected from inoculated mice at different time points during the experiment (**Figure 2**). The changes in antibody levels showed similar trends in all groups. Specifically, during the first 30 days of infection, antibody levels in mice serum increased sharply, reaching the peak level at 60 dpi, and maintained high levels thereafter. Although the antibody levels slightly decreased in all groups from 150 dpi to 270 dpi, they were still greater than those at 30 dpi.

The infection status of the mice was also analyzed via nested PCR (**Table 2**). Unlike the microscopy examination results, nested PCR results showed that all mice were infected with *B. microti* from 2 dpi to 60 dpi. However, at 120 dpi, mice in Groups 1, 3, 4, and 6 were no longer positive for *B. microti*, and only one mouse in Groups 2 and 5 each was still infected. All mice in Groups 1, 3, 4, 5, and 6 were negative for *B. microti* at 150 dpi, and only two were still infected in Group 2. At the end of the experiment (270 dpi), all mice were negative for *B. microti* infection.

**Table 2:** PCR detection of *Babesia microti* infection in BALB/C mice during a 9-month study.

Nested-PCR (No. positive / No. tested)						
Dpi	Group 1 (20%; 5 mice)	Group 2 (4%; 3 mice)	Group 3 (0.8%; 3 mice)	Group 4 (0.16%; 3 mice)	Group 5 (0.032%; 3 mice)	Group 6 (0.0064%; 3 mice)
0						
2	+ (5/5)	+ (3/3)	+ (3/3)	+ (3/3)	+ (3/3)	+ (3/3)
6		+ (3/3)	+ (3/3)			
7	+ (5/5)	+ (3/3)	+ (3/3)	+ (3/3)	+ (3/3)	+ (3/3)
8				+ (3/3)		
10					+ (3/3)	+ (3/3)
14	+ (5/5)	+ (3/3)	+ (3/3)	+ (3/3)	+ (3/3)	+ (3/3)
21	+ (5/5)	+ (3/3)	+ (3/3)	+ (3/3)	+ (3/3)	+ (3/3)
30	+ (5/5)	+ (3/3)	+ (3/3)	+ (3/3)	+ (3/3)	+ (3/3)
45	+ (5/5)	+ (3/3)	+ (3/3)	+ (3/3)	+ (3/3)	+ (3/3)
60	+ (5/5)	+ (3/3)	+ (3/3)	+ (3/3)	+ (3/3)	+ (3/3)
120	- (0/5)	+ (1/3)	- (0/3)	- (0/3)	+ (1/3)	- (0/3)
150	- (0/5)	+ (2/3)	- (0/3)	- (0/3)	- (0/3)	- (0/3)
270	- (0/5)	- (0/2)	- (0/3)	- (0/3)	- (0/3)	- (0/3)

[+]-positive; [-]-negative All mice were negative for B. microti at 270 dpi.

### 3.2 Nested PCR detection limit of *B. microti*

**Figure 3:**
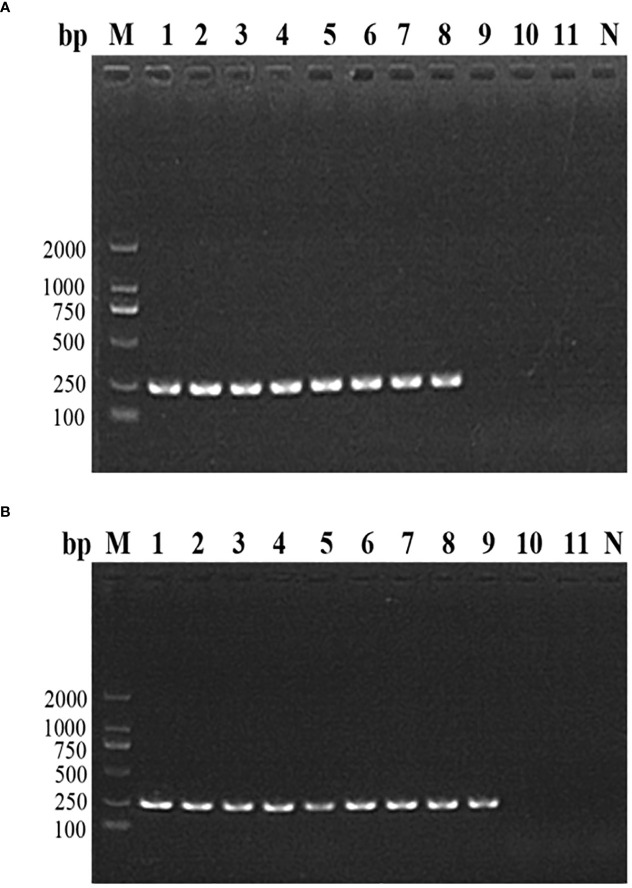
The nested PCR detection limit of *Babesia microti.* **(A)** Nested PCR analysis of 11 *B. microti* DNA samples with different concentrations. The DNA concentration of 3 fg/μL (sample No. 8) was found to be the detection limit of the nested PCR. **(B)** Nested PCR analysis of the DNA of 11 blood samples with different densities of *B. microti*. The blood with a *B. microti* density of 5.48 parasites/μL (sample No. 9) was the detection limit of the nested PCR.

The nested PCR detection limit of the DNA and parasite density of *B. microti* in whole blood was determined by analyzing 11 whole blood samples with an extremely low *B. microti* density (pre-determined different concentrations of pure *B. microti* DNA). The *B. microti* DNA was detected in the 8 samples with *B. microti* DNA concentrations of 30 ng/μL (sample No. 1), 3 ng/μL (sample No. 2), 0.3 ng/μL (sample No. 3), 0.03 ng/μL (sample No. 4), 3 pg/μL (sample No. 5), 0.3 pg/μL (sample No. 6), 0.03 pg/μL (sample No. 7), and 3 fg/μL (sample No. 8) (**Figure 3A**). Moreover, nested PCR could detect *B. microti* DNA in whole blood containing *B. microti* density varying from 2.14 ×10^6^ parasites/μL (sample No. 1), 4.28×10^5^ parasites/μL (sample No. 2), 8.56×10^4^ parasites/μL (sample No. 3), 1.712×10^4^ parasites/μL (sample No. 4), 3.424×10^3^ parasites/μL (sample No. 5), 6.848×10^2^ parasites/μL (sample No. 6), 1.370×10^2^ parasites/μL (sample No. 7), 27.4 parasites/μL (sample No. 8), and 5.48 parasites/μL (sample No. 9) (**Figure 3B**). However, *B. microti* DNA was not detectable in samples 10 and 11, which had *B. microti* densities of 1.09 parasites/μL and 0.22 parasites/μL, respectively. In general, the nested PCR detection limits for the DNA and density of *B. microti* were 3 fg/μL and 5.48 *B. microti* parasites/μL, respectively.

### 3.3 Transmission risk assessment of transfusing blood with extremely low-density *B. microti* in mouse models

**Figure 4:**
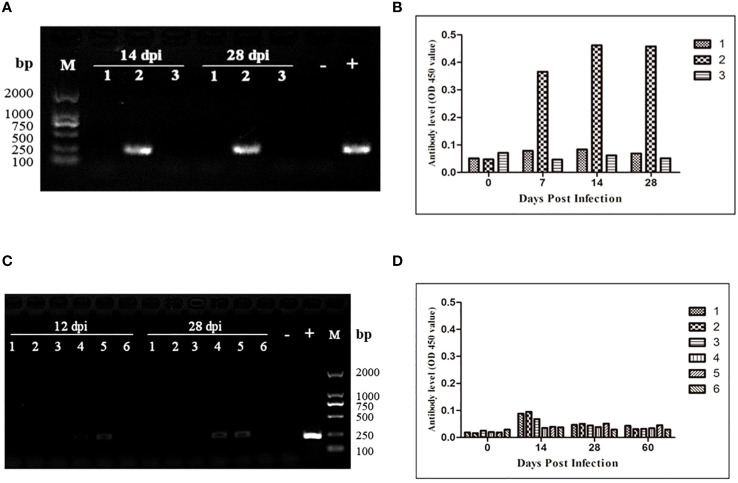
Transmission risk assessment of transfusion blood containing an extremely low density of *Babesia microti* in mouse models. **(A)** Nested PCR detection of the infection status of the BALB/c mice inoculated with whole blood containing *B. microti* at a density of 1.08 parasites/µl. One out of three mice was infected. **(B)** The changes over 28 days in anti-*B. microti* antibody levels in the three mice inoculated. The infected mice produced higher levels of antibodies on 7, 14 and 28 days post-inoculation (dpi). **(C)** Infection status of the second-generation mice inoculated with whole blood collected from the first-generation mice inoculated with blood containing anextremely low density of the *B. microti* (mice in Group 6) for 120 days. **(D)** The changes over 60 days in anti-*B. microti* antibody levels in the three mice inoculated. The antibody levels remained low during the 60 days. All these results confirmed that transfusing blood with an extremely low density of *B. microti* has a high transmission risk.

To assess the transmission risk of transfusion blood with extremely low *B. microti* density, we used whole blood samples containing *B. microti* at a lower density (1.09 parasites/μL) than that of the nested PCR detection limit (5.48 parasites/µL) to inoculate three healthy BALB/c mice. The blood samples of the inoculated mice were collected 28 days after inoculation and analyzed via nested PCR (**Figure 4A**). At 14 and 28 dpi, only one mouse (No. 2) was infected with *B. microti*. The level of anti-*B. microti* antibodies in the blood collected from the inoculated mice were detected with ELISA at different time points during the 28 days. As shown in **Figure 4B**, among the three mice, one mouse (No. 2) always produced the highest amount of antibodies against *B. microti* at 7, 14, and 28 dpi.

According to the nested PCR and microscopic examination used to determine the infection status of the mice inoculated with different densities of *B. microti* (3.1), all the mice inoculated with whole blood at the lowest infection rate (Group 6) for 120 days were not infected with *B. microti*. To evaluate the transmission risk of transfusing the blood of these mice to healthy mice, we collected and transfused their blood into six healthy BALB/c mice. During the 28 days of observation, no *B. microti* was found in the blood of the inoculated mice via microscopic examination; however, nested PCR results showed that 2 of the 6 mice were infected with *B. microti* at 12 and 28 dpi (**Figure 4C**). The ELISA was used to test the level of the specific anti-*B. microti* antibody after 60 days of inoculation, and the results showed that the antibody levels were quite low throughout the 60-day observation period (**Figure 4D**).

### 3.4 Transmission risk assessment of transfusing human blood in mouse models

After the human blood infected with *B. microti* was transfused to healthy mice, the infection status of the inoculated mice was determined using nested PCR and ELISA. Among the 557 human whole blood samples, two *B. microti*-positive blood samples (samples No. 118 and 146) were identified by nested PCR, and among 372 whole blood samples, six blood samples were ELISA-positive for anti-*B. microti* antibodies, among which sample No. 130 had the highest IgG level. After blood sample No. 118 was inoculated into three BALB/c and two NOD-SCID mice, the infection status of these five mice was detected by nested PCR and microscopy examination at different time points (**Table 3**). At 7 and 14 dpi, no mice were infected with *B. microti.* Two BALB/c mice were infected at 21 dpi, while the other mice were not. At 28 dpi, two BALB/c and two NOD-SCID mice were infected. All BALB/c mice were infected at 35 dpi;one NOD-SCID mouse was infected, and the other mouse died due to accidental factors. Moreover, all BALB/c mice and one NOD-SCID mouse remained infected at 84 dpi. However, at 98 dpi, two BALB/c mice and one NOD-SCID mouse were infected. However, during the 98 days, no *B. microti* was identified in the blood of these inoculated mice via microscopic examination.

**Table 3:** Infection status of the mice inoculated with human blood containing *Babesia microti* (No. 118).

Dpi	Nested PCR (No. positive / No. tested)		Microscopy (No. positive / No. tested)	
	BALB/c	NOD-SCID	BALB/c	NOD-SCID
0	–	–	–	–
7	–	–	–	–
14	–	–	–	–
21	+ (2/3)	–	–	–
28	+ (2/3)	+ (2/2)	–	–
35	+ (3/3)	+ (1/2)	–	–
42	+ (3/3)	+ (1/2)	–	–
49	+ (3/3)	+ (1/1)	–	–
56	+ (3/3)	+ (1/1)	–	–
63	+ (3/3)	+ (1/1)	–	–
70	+ (3/3)	+ (1/1)	–	–
77	+ (3/3)	+ (1/1)	–	–
84	+ (3/3)	+ (1/1)	–	–
98	+ (2/3)	+ (1/1)	–	–

[+]-positive; [-]-negative.

Furthermore, 100 µL of whole blood collected from the NOD-SCID mice infected with *B. microti* was inoculated into one healthy NOD-SCID mouse (second generation), and the infection status of these mice was detected via nested PCR and microscopy examination (**Supplementary Data 2.1**). *Babesia microti* was detected in the blood of the mouse at 9 dpi via nested PCR. This infection status was observed via nested PCR at 8 of the 10 pre-determined time points during 65 days. However, no parasites were found via microscopy examination.

After blood sample No. 130 was inoculated into three BALB/c and three NOD-SCID mice, the infection status of these six mice was detected by nested PCR and microscopy examination at different time points (**Table 4**). At 7 and 14 dpi, no mice were infected with *B. microti*. However, one BALB/c mouse and two NOD-SCID mice were infected with *B. microti* at 21 dpi. Two BALB/c mice and one NOD-SCID mouse were infected at 28 dpi, and all mice were infected at 35 dpi. Moreover, this status lasted until 56 dpi. At 63 dpi, all BALB/c mice and two NOD-SCID mice remained positive, while two BALB/c and three NOD-SCID mice were infected at 98 dpi. However, during the 98 days, no *B. microti* was found in the blood of these inoculated mice via microscopic examination.

**Table 4:** Infection status of the mice inoculated with human blood containing *Babesia microti* (No. 130).

Dpi	Nested PCR (No. positive / No. tested)		Microscopy (No. positive / No. tested)	
	BALB/c	NOD-SCID	BALB/c	NOD-SCID
0	–	–	–	–
7	–	–	–	–
14	–	–	–	–
21	+ (1/3)	+ (2/3)	–	–
28	+ (2/3)	+ (1/3)	–	–
35	+ (3/3)	+ (3/3)	–	–
42	+ (3/3)	+ (3/3)	–	–
49	+ (3/3)	+ (3/3)	–	–
56	+ (3/3)	+ (3/3)	–	–
63	+ (3/3)	+ (2/3)	–	–
70	+ (2/2)	+ (2/3)	–	–
77	+ (2/2)	+ (1/3)	–	–
84	+ (2/2)	+ (2/3)	–	–
98	+ (2/2)	+ (3/3)	–	–

[+]-positive; [-]-negative.

Whole blood was collected from the three NOD-SCID mice infected with *B. microti* and mixed, and 100 µL of the mixed blood sample was inoculated into one healthy NOD-SCID mouse (second generation). The infection status of these mice was detected using nested PCR and microscopy examination (**Supplementary Data 2.2**). *Babesia microti* was detected in the blood of the mouse at 9 dpi via nested PCR. This infection status was detected at 6 of the 10 pre-determined time points for the 65 days via nested PCR. However, no parasites were found via microscopy examination.

## 4 Discussion

Babesiosis is a tick-borne disease that has several clinical manifestations. According to the Food and Drug Administration (FDA) data, *Babesia* is the most common pathogen transmitted by blood transfusion (FDA, May 2019). Although the current understanding of diseases caused by this parasitic pathogen is still limited, clinical manifestations in patients with weak immune systems can be extremely severe, and they may require hospitalization when infected with *Babesia*. Therefore, *Babesia* detection should be considered in patients with nonspecific symptoms, a history of blood transfusion, or possible tick exposure in relevant endemic areas (Strizova et al., 2020).

This study investigated the infection progress of mice inoculated with blood containing different densities of *B. microti* (at different infection rates) and found that the infection rate significantly affected the infection peak of the inoculated mice. A high infection rate in blood resulted in an early infection peak. However, the level of anti-*B. microti* antibodies produced in all inoculated mice showed no significant difference. Mice in all groups produced high levels of anti-*B. microti* antibodies after infection, and this high antibody level was maintained for a longer period (up to 270 dpi). This result suggested that the blood containing extremely low *B. microti* density can still stimulate the host to produce high concentrations of antibodies, indicating the necessity and feasibility of screening for specific antibodies against *B. microti* in high-risk populations.

The detection of antibody levels is important for the screening of latent infection cases. In the study of the protective immune mechanism of *B. microti* infection, it was shown that during the acute infection stage, specific IgM antibodies were first produced in response to parasites, while specific IgG antibodies were related to the reduction of parasites. However, during infection, the immune response spectrum from the acute phase to the chronic phase remains unclear. Thus, it is of great value to find biomarkers indicating the disease progress and the antigens involved in immune protection.

This study confirmed that the nested PCR test is limited by the density of the parasites in the blood and relies on a detection limit (5.48 parasites/μL), which could be important when used in a clinical setting. It was confirmed that the blood containing an extremely low-density *B. microti* (1.09 parasites/μL) still had a high transmission risk via blood transfusion. This study also confirmed that even the blood collected from the mice inoculated for 120 days with blood containing an extremely low-density of *B. microti*, which tested negative for *B. microti*, could still transmit *B. microti* to healthy mice (**Figure 4**). Wang et al. (2015) reported that the limit of detection of real-time PCR assays was approximately 1-3 parasites/μL of blood. Therefore, our study covered the limit of detection of a real-time PCR assay (Wang et al., 2015).

Moreover, this study also found that the level of anti-*B. microti* antibodies produced in the mice that were initially infected with blood containing extremely low-density *B. microti* for 120 days (**Figure 2**) were higher than those produced in the mice infected with *B. microti* transmitted by the whole blood collected from the first-generation mice (**Figure 4D**). This may be due to the difference in the immune responses of the host to the whole blood stimulus from different sources and the vitality of the *B. microti* in different blood samples. At 120 dpi, the inoculated mice produced higher levels of antibodies against *B. microti* (**Figure 2**). When the host produces higher levels of antibodies against parasites, the activity of the parasites in the host is usually inhibited (Wang et al., 2016). Therefore, when the blood of the inoculated mice was transfused to healthy mice, the *B. microti* with inhibited function could not stimulate high-level production of antibodies in the new host. However, when the blood was diluted to a lower density of *B. microti*, the parasites could reproduce as the host had not yet produced high enough levels of anti-*B. microti* antibodies. Therefore, when these parasites infect a new host again, they can promote the production of higher antibody levels (Cai et al., 2018).

In this study, 557 human blood samples were tested, of which eight samples were positive for *B. microti*, indicating a positive rate of 1.43%. Moritz et al. (2016) tested 89153 blood donation samples between 2012 and 2016, of which 335 samples tested positive for *B. microti*, with a positive rate of 0.38%. When 46 *B. microti*-positive blood samples identified with PCR tests were inoculated into hamsters, 25 blood samples (54%) could transmit *B. microti* to the hamsters. In addition, 47 blood samples were *B. microti*-negative for the PCR tests but positive for the AFIA (≥ 1:512). When the red blood cells of these samples were inoculated into hamsters, only two samples could transmit the *B. microti* to hamsters. The current study also confirmed that positive blood samples could transmit *B. microti* to new hosts, revealing a high transmission risk of blood infected with *B. microti*. These results suggest that it is necessary to screen blood donations for *Babesia* to avoid transmission through blood transfusion. *Babesia* can be transmitted through blood transfusion and cause severe life-threatening hemolytic anemia in high-risk patients, such as sickle cell disease patients. The rarity of diagnosis, similar clinical manifestations, and delayed hemolytic transfusion reaction may lead to delayed diagnosis and inappropriate treatment using steroids or other immunosuppressive drugs. The incidence rate of babesiosis, particularly in vulnerable populations, proves the necessity of implementing a universal blood screening program in endemic areas (Karkoska et al., 2018). In addition, the transfusion of blood containing *Babesia* may not necessarily cause obvious clinical symptoms, indicating the possibility of many latent carriers. This latent carrier group should not be ignored in clinics as *Babesia* can be a potential threat to their health.

The disease spectrum of babesiosis is wide, with significant differences in severity from surface silent infections to opportunistic deaths caused by malaria-like symptoms, making it highly susceptible to misdiagnosis or missed diagnosis in clinical practices (Gelfand and Callahan, 2003; Vannier and Krause, 2009). In the United States, most babesiosis cases are caused by infection with *B. microti*. It has been reported that between 1979 and 2009, 159 out of 162 blood transfusion-related babesiosis cases were identified as being caused by *B. microti* (Moritz et al., 2014). In a study of entombemia and immune reaction dynamics using rhesus monkeys as an experimental animal model, an extremely low infection dose of *B. microti* was shown to cause blood transfusion infection in *Babesia*, and the asymptomatic hypoentombemia state after infection lasted for a long time (Ruebush, 1980; Sanjeev et al., 2016). According to a screening study of *B. microti* in blood donors in the United States, 97.3% of blood donors who tested positive for the pathogen were also antibody-positive (Moritz et al., 2016). However, 80% of blood donors were negative for *B. microti* infection when subjected to nested PCR tests but positive when subjected to antibody tests. These individuals were confirmed to still have a risk of transfusion transmission when they donated their blood to others. In a follow-up treatment and testing of *Babesia* patients, it was also found that higher levels of the specific antibodies against *B. microti* could last for a considerable period, ranging from a few months to over a year or even longer (Hildebrandt et al., 2007; Knapp and Rice, 2015). Therefore, clinicians should improve their understanding of babesiosis, and further research is needed to establish the best management of this disease in high risk groups (including HIV-infected patients and blood donors) (Bondarenko et al., 2021).

## 5 Conclusion

This study shows that the mice were inoculated with blood containing different densities of *B*. *microti* (at different infection rates), and the infection rate significantly influenced the infection peak in the inoculated mice. However, the highest level of anti*-B. microti* antibodies produced in each group of mice showed no significant difference. Most importantly, when transfused between mice and mice or humans andmice, whole blood containing an extremely low density of *B. microti* still poses a high transmission risk during blood transfusion.

## 6 Limitations

Our study also had several limitations. First, the design was retrospective, leading to the exclusion of some data and interpretation difficulties. Second, we used different numbers of mice in the experiment, especially in the second generation of the human-to- mouse model, where we used one NOD-SCID mouse, possibly causing experimental bias. A third limitation is that the selection of the study population and blood donors might have been more persuasive. Fourth, more sensitive and higher quality data could have been generated if real-time PCR was used.

## Data availability statement

The original contributions presented in the study are included in the article/**Supplementary Material**. Further inquiries can be directed to the corresponding authors.

## Ethics statement

The studies involving humans were approved by the Medical Ethics Committee of National Institute of Parasitic Diseases, Chinese Center for Disease Control and Prevention (Chinese Center for Tropical Diseases Research) (Ethical approval number 2019004). The studies were conducted in accordance with the local legislation and institutional requirements. The participants provided their written informed consent to participate in this study. The animal study was approved by The Laboratory Animal Welfare & Ethics Committee (LAWEC) of the Chinese Centre for Tropical Diseases Research and Shang Hai Blood Center approved the animal experiments (permit Number: IPD-2019-14). The study was conducted in accordance with the local legislation and institutional requirements.

## Author contributions

YCC: Conceptualization, Methodology, Writing – original draft. BX: Conceptualization, Methodology, Writing – original draft. XFL: Methodology, Data curation, Formal analysis. WWY: Methodology, Data curation, Formal analysis. ZRM: Data curation, Formal analysis. ZB: Supervision, Funding acquisition, Writing – review & editing. JXC: Supervision, Funding acquisition, Writing – review & editing. WH: Conceptualization, Supervision, Funding acquisition, Writing – review & editing. All authors agreed to the final manuscript.
