# Supplementary material for: Transmission risk evaluation of transfusion blood containing low-density Babesia microti
Source: Front Cell Infect Microbiol. 2024 Feb 5;14:1334426. doi: 10.3389/fcimb.2024.1334426 (PMC10875030; doi:10.3389/fcimb.2024.1334426)
Supplement: Supplementary file 1 [file DataSheet_1.docx]

**Supplementary data 1**

Screening for *B. microti* DNA in human blood samples using nested PCR.


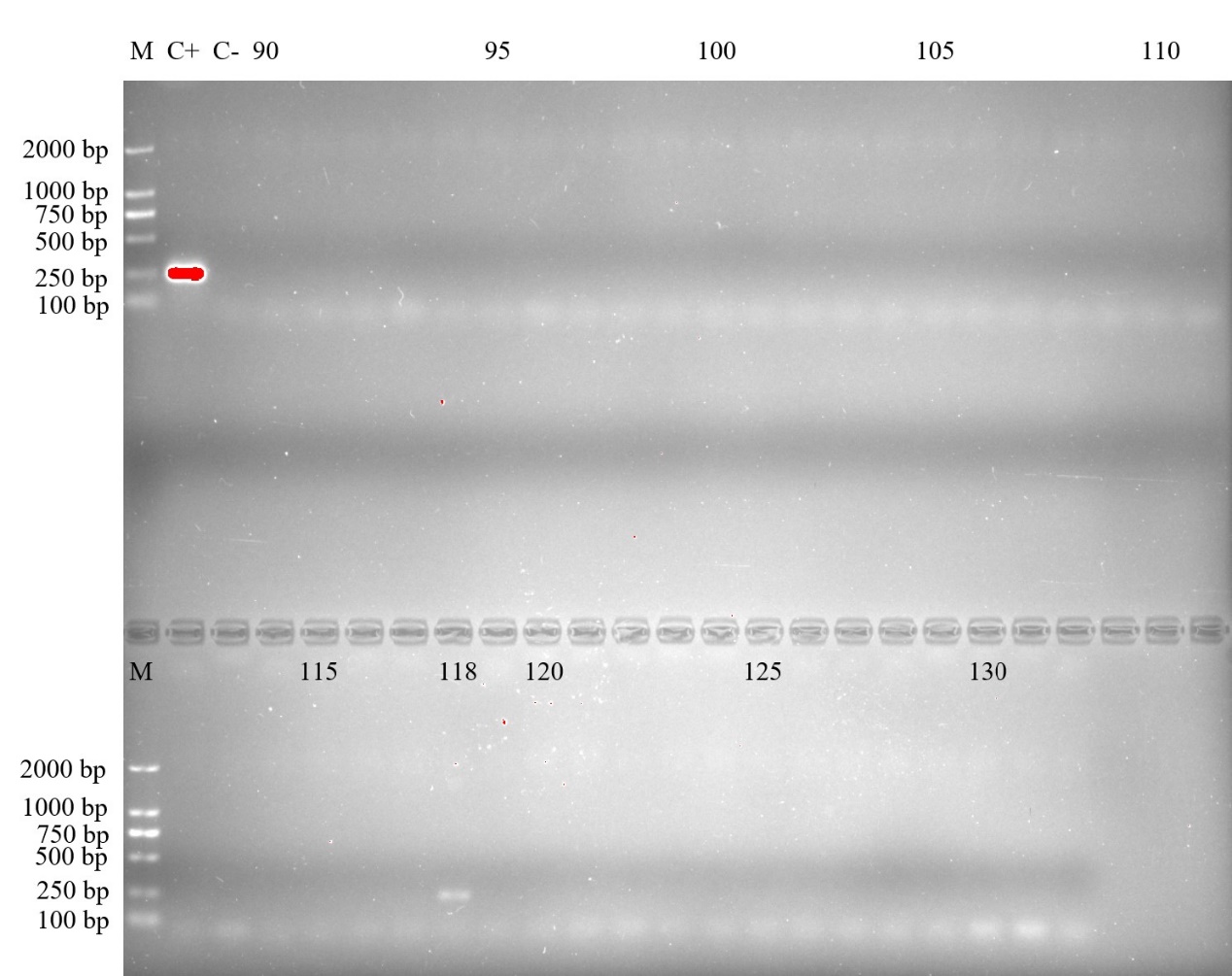


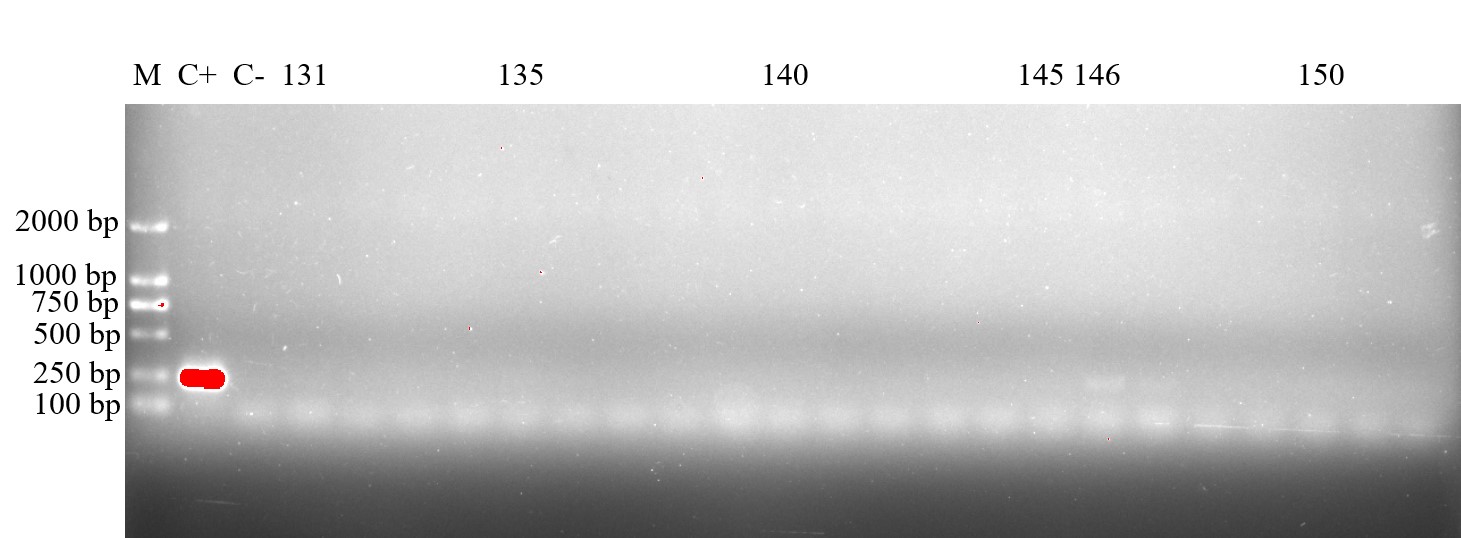


M: marker C+: positive control C-: negative control

Sample “118” and “146” were screened as PCR-positive.

**Screening for *B. microti* antibodies in human blood samples using ELISA.**

Sample“130”


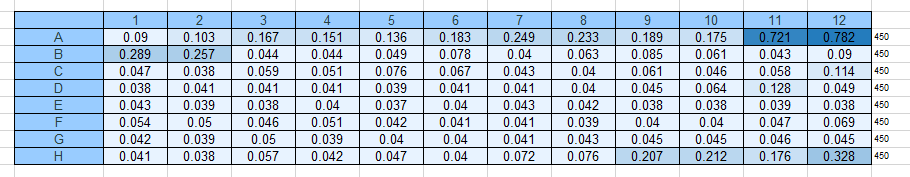


Sample “130” had highest IgG level among all 6 positive samples identified by ELISA
