# Supplementary material for: Transmission risk evaluation of transfusion blood containing low-density Babesia microti
Source: Front Cell Infect Microbiol. 2024 Feb 5;14:1334426. doi: 10.3389/fcimb.2024.1334426 (PMC10875030; doi:10.3389/fcimb.2024.1334426)
Supplement: Supplementary file 2 [file DataSheet_2.docx]

**Supplementary data 2**

2.1 Infection status of a NOD-SCID mouse inoculated intraperitoneally with 100 µL of mixed blood sample collected from the mice inoculated with the human blood sample No.118.

[+]-positive, [-]-negative

2.2 Infection status of a NOD-SCID mouse inoculated intraperitoneally with 100 µL of mixed blood sample collected from the mice inoculated with the human blood sample No.130. The infection status of mice in all experiments were monitored using Nested PCR and microscopy examination.

[+]-positive, [-]-negative
